# Textbook–Atlas of Intestinal Infection in AIDS

**DOI:** 10.3201/eid1101.040986

**Published:** 2005-01

**Authors:** Martin J. Blaser

**Affiliations:** * New York University School of Medicine, New York, New York, USA

**Keywords:** Atlas of Intestinal Infection in AIDS, Daniele Dionisio

Gastrointestinal tract infections are prominent in patients with AIDS. Infections may be caused by a variety of bacterial, fungal, viral, protozoal, and helminthic pathogens, and affect persons in both developing and industrialized countries. The problems are especially acute in resource-limited countries where little or no access to highly active antiretroviral therapy exists; the impact of illnesses associated with HIV is most pronounced in these countries.

Daniele Dionisio, an authority in parasitology, has assembled a new treatise, Textbook–Atlas of Intestinal Infections in AIDS, that directly addresses this topic. In 489 pages, the work includes much background, including a fascinating chapter by Esther Diane on the history of the discovery of intestinal parasites. The work and illustrations of parasitic life cycles by Dionisio and colleagues are illuminating for all students of infectious diseases.

Much of the book addresses particular agents and the diseases they cause. A particular strength is the numerous illustrations. Although varying in quality, they represent an enormous compendium of information about these common problems. The figures on microscopic and ultra-structural pathology are particularly strong.

This volume should be considered as a background text for the pathologist, gastroenterologist, or infectious disease specialist who concentrates on HIV infections. The illustrations and references provide readers access to materials not easily obtained. The sections on clinical management of particular conditions are relatively sparse, and healthcare workers with patients with active problems should consult with a more comprehensive text. In total, this textbook-atlas is a useful addition in the battle against HIV infections and its complications. The editor and multinational group of authors are to be congratulated on their scholarly work.

